# Modeling and Experimental Analysis of Hybrid Cantilever Structures with Embedded MFC Patch

**DOI:** 10.3390/ma18194610

**Published:** 2025-10-05

**Authors:** Andrzej Mitura

**Affiliations:** Department of Applied Mechanics, Lublin University of Technology, Nadbystrzycka 36, 20-618 Lublin, Poland; a.mitura@pollub.pl

**Keywords:** electromechanical model, macro fiber composite transducer, electromechanical coupling estimation, experimental research

## Abstract

This study presents the modeling and analysis of a composite structure incorporating an embedded macro fiber composite (MFC) patch. MFC actuators are available in several variants, with types P1 and P2 being the most commonly used. In this paper, an electromechanical model of the hybrid structure is developed, and experimental procedures are outlined for identifying selected system parameters. In the first phase of the study, two separate cantilever beam specimens are investigated—one with an embedded P1 patch and the other with a P2 patch. Their behaviors are tested and compared to identify and critically assess the advantages and limitations associated with each MFC type. In the second phase, a more complex system—a bistable cantilever shell—is examined. The choice of the appropriate MFC type (P1 or P2) for this structure is based on the findings obtained in the first phase. For the system incorporating the selected MFC patch, the dynamic response is analyzed in the vicinity of both stable equilibrium states, which are characterized by significantly different levels of pre-strain and pre-stress. The study concludes with highlights for the design of smart composite structures with integrated MFC patches.

## 1. Introduction

A hybrid structure can be defined as a combination of different materials or components connected to achieve optimal or adaptive properties. In the literature, particular attention has been given to multilayer hybrid structures. Typically, the core of such a system is a layered composite, within which conventional or smart materials are incorporated either between or outside the layers. In classical applications, fiber-reinforced composites with full or partial metallic layers are often employed. Such modifications aim to enhance specific structural characteristics. For example, in [1], glass–epoxy and carbon–epoxy composites with additional titanium layers were investigated. The incorporation of metallic layers was intended to control structural deformation during low-velocity impacts. The study demonstrated that this modification significantly influenced damage propagation. Another example of a conventional modification is the inclusion of metal strips within bistable structures [2]. Aluminum strips embedded in a carbon–epoxy shell altered the curvature of the structure and affected the force required for snap-through behavior. Before moving on to the target group of hybrid structures, it is also necessary to mention multilayer structures, in which measuring elements (sensors), such as strain gauges, thermocouples, and others, can be embedded into the structure. These sensors can be used, for example, to monitor the condition of the structure during operation or, as shown in [3], already at the production stage. More advanced modification capabilities can be achieved using smart layers. Additional materials with controllable properties include magnetorheological fluids, piezoelectric elements, and shape memory alloy (SMA) fibers. Magnetorheological fluids can vary their apparent viscosity in response to a magnetic field, which can be utilized to increase damping in hybrid structures. In [4], a three-layer beam was studied in which the middle layer was filled with a magnetorheological fluid. A comparison of the beam’s vibrations with and without an applied magnetic field showed that activating the fluid effectively reduced structural vibrations. However, this approach provides only passive or semi-active control by dissipating energy. Greater functionality is offered by shape memory alloy fibers, which can change stiffness and shape in response to temperature. Research on laminated composite plates embedded with SMA wires is presented in [5], where the authors analyzed the influence of temperature on natural frequencies and the thermal buckling behavior of the hybrid structure. Piezoelectric materials appear to offer the most versatile application potential, as they enable both vibration control and energy harvesting. An example of how fiber orientation affects the dynamic behavior of such a structure is found in [6]. However, embedding individual piezoelectric fibers poses significant manufacturing challenges. A more practical solution involves using prefabricated piezoelectric patches. One of the leading manufacturers of such elements is Smart Material Corp. [7], which produces macro fiber composite (MFC) patches. MFC patches feature a complex internal structure, where the electrode configuration, in addition to the piezoelectric fibers themselves, plays a crucial role. A key advantage is their high flexibility, which allows integration without significantly altering the host structure’s stiffness. Depending on the electrode configuration, MFCs can generate an electric field either along or across the patch. This distinction gives rise to two main types: P1, which utilizes the d33 piezoelectric effect, and P2, which utilizes the d31 effect. These types differ not only in the direction of the induced electric field but also in capacitance and voltage characteristics. According to the manufacturer, P1-type elements are intended primarily for vibration control, while P2-type elements are designed for energy harvesting. An application of MFC-P1 patches for vibration control is presented in [8], where MFCs were installed on composite rotor blades. Applying voltage to the patches resulted in measurable changes in the rotor’s natural frequencies. A contrasting application is described in [9], where an MFC-P2 patch was attached to a cantilever beam. As the beam deformed during vibration, the patch generated voltage via the direct piezoelectric effect. However, the findings in [10] depart from these general guidelines. In this experimental study, a bistable shell was tested, where the snap-through phenomenon led to significant changes in the equilibrium position, accompanied by large strains in the MFC patch. The authors observed that P1 elements performed more reliably under these demanding conditions, while P2 elements were more prone to damage. These findings highlighted the need for a direct comparison of the performance of P1 and P2 types under similar conditions—an area insufficiently explored in the current literature.

In the present work, two types of cantilever composite structures with embedded MFC patches were investigated. The first configuration involved two identical cantilever composite beams—one with an MFC-P1 patch and the other with an MFC-P2 patch. Experimental studies examining both the direct and inverse piezoelectric effects enabled a comparative evaluation of the effectiveness of each element, highlighting their respective advantages and limitations. The aim of this part of the research was to contribute to the current state of knowledge by providing a clear comparison of both types of MFCs working under similar conditions. The second configuration consisted of a bistable cantilever composite shell equipped with an MFC-P1 patch. In this case, experiments revealed differences in MFC performance during vibrations around the two stable equilibrium states, corresponding to different initial stress/strain states in the patch. The study of multistable structures is a popular topic within the scientific community. An important aspect of this work is demonstrating the relationship between electromechanical coupling and bistable shell vibration modes. In addition, a lumped-parameter model of the hybrid structure was developed, incorporating both mechanical and electrical domains. This model was used to analyze the system’s basic characteristics and to illustrate the parameter identification procedures. Developing an accurate mathematical model of a multistable structure with an embedded MFC element is complex and time-consuming. Reduced models offer a practical alternative. From this perspective, developing procedures for identifying a surrogate model appears worthwhile. Therefore, this paper proposes procedures to evaluate selected modeling issues related to the reduced-order model. The combined experimental and modeling approach provides insights into the capabilities of MFC-based hybrid structures for both vibration control and energy harvesting applications.

## 2. Electromechanical Model of Hybrid Structure

**Figure 1:**
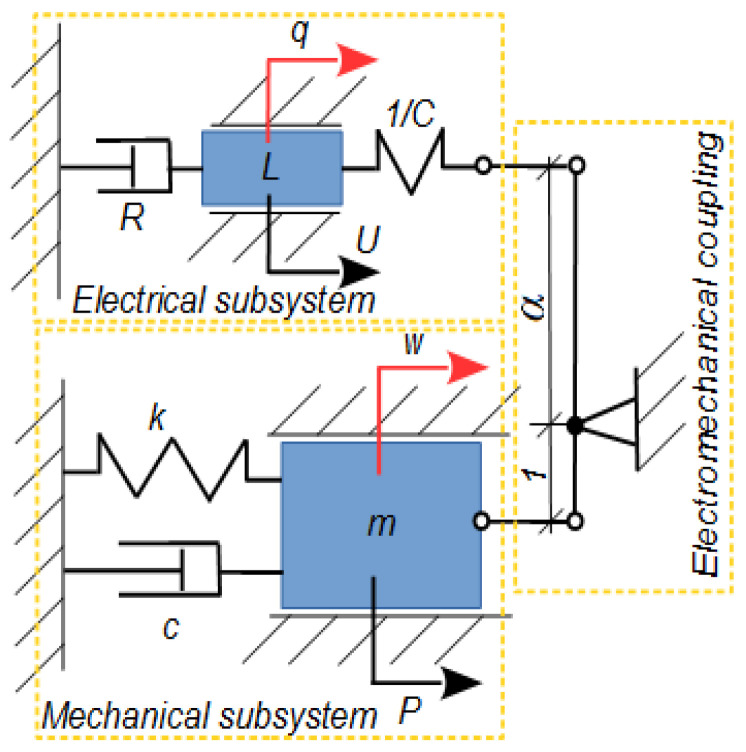
Schematic of the lumped electromechanical system model.

By adding a piezoelectric layer to the structure, an electromechanical system is obtained. A lumped mass model of such a system is presented in Figure 1. In this schematic, two subsystems—mechanical and electrical—along with the coupling mechanism between them, can be distinguished. The diagram serves as a mechanical analogy of the electromechanical structure. The first mechanical subsystem represents a simplified model of a cantilever structure. The parameters included are follows:

- *k*, *c*, and *m*—representing the equivalent stiffness, damping, and mass, respectively;
- *P*—the external excitation force;
- *w*—the transverse displacement at a selected point of the structure, such as its free end.

The second subsystem describes the electrical domain, reflecting both the inherent properties of the piezoelectric patch and any additional components (e.g., amplifier or energy harvester). The generalized coordinate q denotes the electric charge, or equivalently, the current in the circuit. The primary electrical parameters include the following:

- *L*—inductance;
- *C*—capacitance of the piezoelectric element;
- *R*—load resistance (representing the harvesting element);
- *U*—applied external voltage (representing an amplifier or controller output).

The two subsystems are coupled. In the mechanical analogy, this coupling is represented by a lever mechanism [11]. The lever has two arms of lengths 1 and *α*, where *α* is the electromechanical coupling coefficient. The coupling is considered to operate within the linear range. The resulting lumped model can be described by a set of coupled differential equations [12]:

(1a) $$m\ddot{w}+kw+c\dot{w}+\alpha \frac{1}{C}\left(\alpha w-q\right)=P,$$

(1b) $$L\ddot{q}+R\dot{q}-\frac{1}{C}\left(\alpha w-q\right)=U.$$

Due to the typically small values of *L* and *C*, simplifications are applied. Specifically, the inductance *L*, appearing in the numerator, is neglected, while the capacitance *C*, appearing in the denominator as 1/*C*, contributes significantly and is retained. Furthermore, Equation (1a) is normalized by dividing by mass *m*. The simplified equations are then expressed as follows:

(2a) $$\ddot{w}+\omega_{o}^{2}w+2n\dot{w}+\alpha_{1}\frac{1}{C}\left(\alpha w-q\right)=P^{*},$$

(2b) $$R\dot{q}-\frac{1}{C}\left(\alpha w-q\right)=U.$$

where

- *ω_o_*^2^ = *k*/*m*—natural frequency;
- 2*n* = *c*/*m*—damping ratio;
- P* = *P*/*m*—mechanical force factor;
- *α*_1_ = *α*/*m*—additional parameter related to the coupling.

As mentioned earlier, either an amplifier or an energy harvester can be connected to the piezoelectric element. When only the amplifier is connected, the piezoelectric element operates as an actuator (i.e., *R* = 0 Ω). If the applied voltage *U* is constant, it induces a steady displacement *w*. In this case, the simplified form of Equations (2a) and (2b) becomes

(3a) $$\omega_{o}^{2}w=-\alpha_{1}\frac{1}{C}\left(\alpha w-q\right)=\alpha_{1}U,$$

(3b) $$U=-\frac{1}{C}\left(\alpha w-q\right).$$

The electromechanical coupling coefficient *α*_1_ can be determined from the relationship:

(4) $$\alpha_{1}=\frac{\omega_{o}^{2}w}{U}.$$

In summary, the parameter *α*_1_ can be estimated from a straightforward experimental procedure (referred to as TEST 1). A constant voltage *U* is applied, and the resulting steady-state displacement *w* is measured. Additionally, the natural frequency *ω_o_* of the mechanical subsystem must be known.

In contrast, when only the energy harvester is connected, the external voltage *U* is zero. In this case, Equation (2b) simplifies to

(5) $$R\dot{q}+\frac{1}{C}q=\frac{\alpha }{C}w.$$

This represents a first-order linear ordinary differential equation. Assuming a harmonic mechanical excitation of the form *w* = *w_o_*sin*ωt*, the amplitude of the resulting electric charge *q_o_* is given by

(6) $$q_{o}=\frac{\alpha w_{o}}{\sqrt{1+R^{2}C^{2}\omega^{2}}}.$$

The amplitude of the current flowing in the circuit is then

(7) $${\dot{q}}_{o}=q_{o}\omega =\frac{\alpha w_{o}}{\sqrt{\frac{1}{\omega^{2}}+R^{2}C^{2}}}.$$

Consequently, the voltage amplitude across the load resistor is

(8) $$U_{R}=R{\dot{q}}_{o}=\frac{\alpha w_{o}}{\sqrt{\frac{1}{\omega^{2}R^{2}}+C^{2}}}.$$

From Equation (8), the coupling coefficient α can be extracted as follows:

(9) $$\alpha =\frac{U_{R}\sqrt{\frac{1}{\omega^{2}R^{2}}+C^{2}}}{w_{o}}.$$

Based on Equation (9), a second experimental procedure (TEST 2) can be designed. Under periodic excitation at frequency ω, the displacement amplitude *w_o_* of the mechanical system and the voltage amplitude *U_R_* across the load resistor should be measured. In addition, the values of the load resistance *R* and the capacitance *C* must be known. By performing TEST 1 (Equation (4)) and TEST 2 (Equation (9)), the coupling coefficients *α*_1_ and *α* can be experimentally determined. These coefficients are subsequently used in the experimental analysis and theoretical modeling presented in the following sections.

## 3. Study of a Cantilever Beam with an Embedded MFC Patch

**Figure 2:**
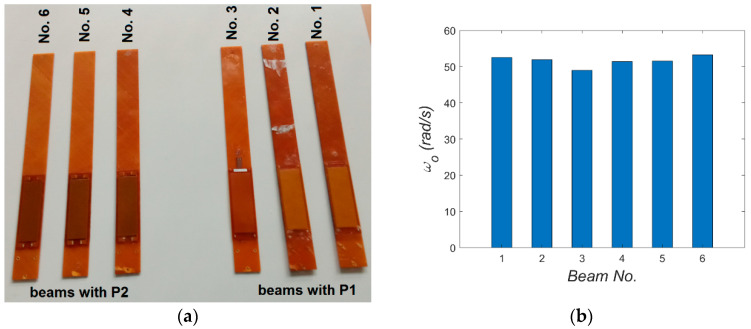
Photo of the beams (**a**) and their first bending natural frequency (**b**).

The research object taken for the tests comprised six-layer beams ([±45/90]_S_) made from a glass–epoxy laminate. The material properties and geometric dimensions of the beams are detailed in [8]. Specifically, a composite square plate with a side of about 90 cm was produced in an autoclave, from which two sets of beams were cut. The beams were cut by machine (water jet cutting) from the central part of the plate, where the thickness has the smallest deviations. In principle, all beams were intended to have identical properties, including geometry, with cantilever length, width, and thickness equal to 316 mm, 34 mm, and 1.8 mm, respectively. Subsequently, M8528-P1 or M8528-P2 patches were bonded onto the beams, resulting in two sets: three beams with P1 patches and three beams with P2 patches (see Figure 2a). Using modal analysis, the first bending natural frequencies of all beams with embedded MFC elements were compared. During these tests, the beams were clamped in such a way that the engagement of clamping elements remained in each test. The beam was excited using the PCB 086D80 modal hammer, and its response was recorded by the PCB 352A24 accelerometer attached to its end. Both signals were analyzed using Scadas Mobile analyzer and Simcenter TestLab v2021 software. The obtained results are summarized in Figure 2b. Modal analysis confirmed that the properties were consistent across samples, with the error in the first natural frequency not exceeding 8%. Beams with the closest natural frequencies were selected for comparative studies. The choice fell on beams No. 2 with element P1 and No. 5 with element P2. Two types of experiments (TEST 1 and TEST 2) were conducted on these beams exhibiting the closest mechanical properties.

**Figure 3:**
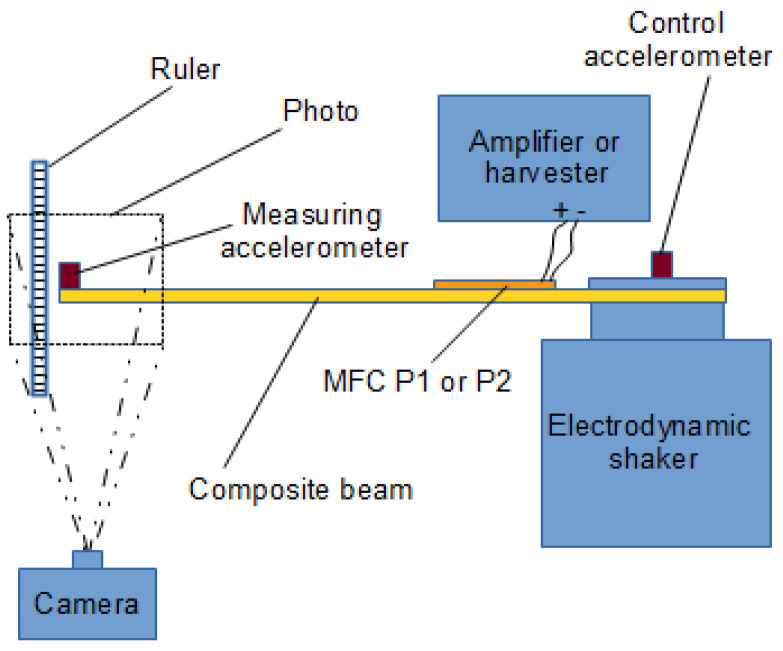
Schematic of the experimental setup.

A schematic of the test setup is shown in Figure 3. The right side of the schematic depicts the amplifier (used in TEST 1), the electrodynamic shaker, and the harvester (used in TEST 2). The left side illustrates the method of measuring the beam response. During the first static test, the deflection of the beam tip was measured under the application of a constant voltage *U* to the MFC patch (actuator mode). The constant voltage from the signal generator was amplified (1:200) by the HVA1500/50-3 amplifier and used to power the piezoelectric element, which generates the beam’s deformation. The deflection was recorded using the D5000 Nikon camera (Nikon Corporation, Tokyo, Japan), and the applied voltage was monitored via the AX-18B Axiomet multimeter at the amplifier input. The camera images were analyzed using Tema v3.8 software, and a ruler was used as a reference for pixel-to-millimeter scaling. In the second, dynamic test, the beam vibrations were excited using the Tira TV50101 electrodynamic shaker, which induced voltage in the MFC patch (harvester mode). The voltage across the harvester resistor *U_R_* was recorded after using a voltage divider (5:1 or 25:1) through the voltage input of Scadas Mobile, while the beam vibrations were measured with the PCB 352A24 accelerometer. The electrodynamic shaker was controlled in a closed-loop feedback system, using a second PCB 352A24 accelerometer glued to the shaker armature as the control sensor *a_shaker_*(*t*). Since the accelerometer measures absolute acceleration *ẅ_absolute_*(*t*), but the relative acceleration *ẅ_relative_*(*t*) is required for calculations, the following relation applies [13]:

(10) $$\begin{array}{l} {\ddot{w}}_{relative}\left(t\right)={\ddot{w}}_{absolute}\left(t\right)-a_{shaker}\left(t\right),\\ or\\ {\ddot{w}}_{o}\sin\left(\omega t+\phi_{o}\right)={\ddot{w}}_{a}\sin\left(\omega t+\phi_{a}\right)-a_{s}\sin\left(\omega t\right), \end{array}$$

where *ẅ_o_*, *ẅ_a_*, and *a_s_* denote the relative beam acceleration, absolute beam acceleration, and shaker acceleration amplitudes, respectively. By performing appropriate trigonometric transformations, the amplitude of relative vibrations can be determined:

(11) $${\ddot{w}}_{o}^{2}={\ddot{w}}_{a}^{2}-2{\ddot{w}}_{a}a_{s}\cos\left(\phi_{a}\right)+a_{s}^{2},$$

and at the resonance frequency (*φ_a_* = *π*/2)

(12) $${\ddot{w}}_{o}^{2}={\ddot{w}}_{a}^{2}+a_{s}^{2}.$$

This relation enables the determination of the beam vibration amplitude required for further analysis.

### 3.1. Application of MFC as an Actuator

**Figure 4:**
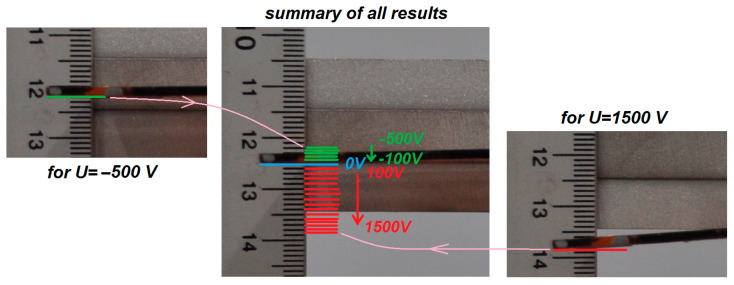
Experimental results for the beam with P1 patch.

**Figure 5:**
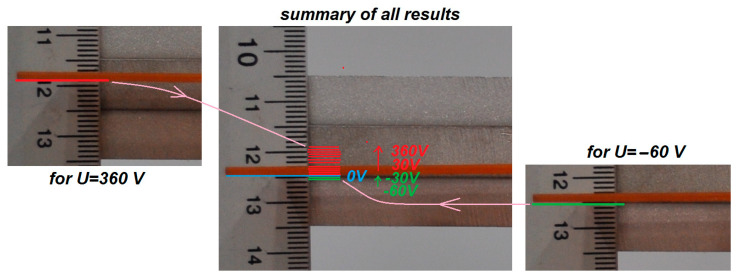
Experimental results for the beam with P2 patch.

In TEST 1, the voltage applied to the piezoelectric elements P1 or P2 was incrementally varied using an amplifier. These elements operate over different voltage ranges: from −500 V to +1500 V for type P1, and from −60 V to +360 V for type P2 [7]. During the tests, the voltage was increased from minimum to maximum in steps of 100 V for P1 and 30 V for P2. At each voltage level, photographs of the beam tip position were taken. To facilitate accurate measurements, a ruler was positioned near the beam tip, serving as a reliable reference scale. A series of photographs was collected for both types of elements. The paper presents one representative photograph each for the systems with P1 and P2 patches, showing the equilibrium position at 0 V in the middle image in Figure 4 and Figure 5. Horizontal lines indicating beam tip positions in the next photos were added to these images with 0 V, while vertical arrows denote the direction of the amplifier voltage change (red lines − positive voltages, green lines − negative voltages). In addition, the maximum and minimum beam tip position at extreme voltages *U* is visualized in the right and left sides of Figure 4 and Figure 5.

**Figure 6:**
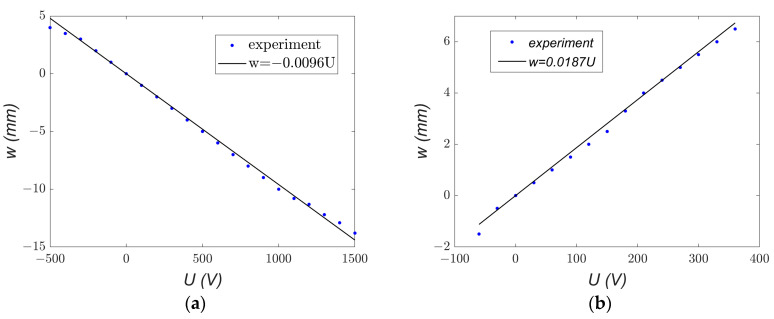
Experimental and analytical trends of displacement *w* as a function of applied voltage *U* for (**a**) MFC-P1 and (**b**) MFC-P2.

Analysis of Figure 4 and Figure 5 reveals that, for the same voltage polarity, the beams deform in opposite directions: under positive voltage, the beam with the P1 element deflects downward, whereas the beam with the P2 element moves upward. This difference is attributed to the distinct piezoelectric mechanisms associated with the d33 and d31 effects. Using Equation (4) and applying the least squares method to the measurement data, the values of the coupling coefficient *α*_1_ were determined for both elements. The natural frequency value *ω_o_* = 51.71 rad/s, taken from Figure 2b, was used in the calculations. Figure 6 illustrates the experimental relationship *w* = *f*(*U*) along with its linear approximation. 

From Equation (4), the *α*_1_ coefficients were calculated as −25.67 × 10^−3^ ms^−2^V^−1^ for MFC-P1 and 50.002 × 10^−3^ ms^−2^V^−1^ for MFC-P2. Comparing the two MFC types reveals that the *α*_1_ coefficient for P2 is more than twice as large as that for P1, indicating that P2 is more effective in producing beam deformation per unit voltage. However, P2 exhibits a significant disadvantage: a smaller range of achievable displacement. Considering the displacement range |*w_max_ − w_min_*| observed in Figure 6, the P1 type showed more than twice the displacement range of P2 (approximately 17 mm versus 8 mm). This highlights a clear conflict between actuation effectiveness and the operational displacement range for the two types of MFC elements.

### 3.2. Application of MFC as a Harvester

**Figure 7:**
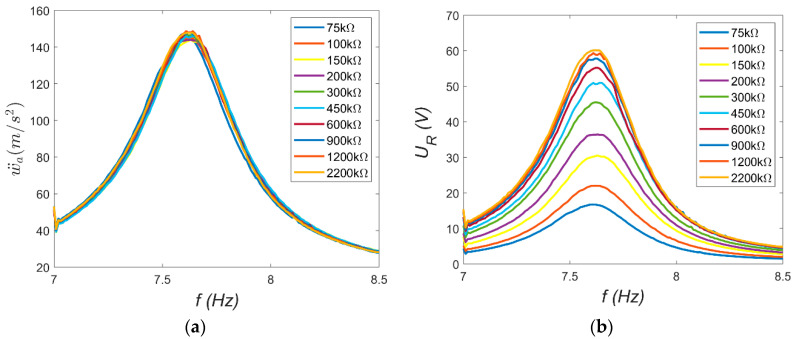
Resonance characteristics for MFC-P1: (**a**) absolute beam vibration amplitude; (**b**) resistor voltage amplitude.

**Figure 8:**
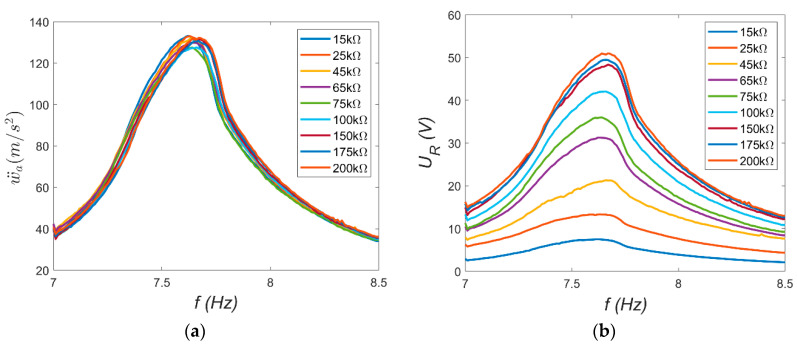
Resonance characteristics for MFC-P2: (**a**) absolute beam vibration amplitude; (**b**) resistor voltage amplitude.

In TEST 2, the electrodynamic shaker and the harvester played key roles. The shaker generated a kinematic excitation with constant acceleration amplitude *a_s_* = 0.4 g, while the frequency *f* was swept slowly and linearly from 7 Hz to 8.5 Hz. The harvester consisted of a combination of resistors used to vary the load resistance *R* of the electrical circuit. A series of measurements was conducted with different load resistances: from 75 kΩ to 2200 kΩ for MFC-P1 and from 15 kΩ to 200 kΩ for MFC-P2. The resonance characteristics of the absolute vibration amplitude at the beam tip *ẅ_a_* and the voltage across the resistor *U_R_* were recorded using the Scadas Mobile analyzer. The resulting characteristics are shown in Figure 7 and Figure 8.

Several observations can be made based on the resonance characteristics. First, the beam’s resonance frequency *ω_r_* can be estimated from the frequency corresponding to the maximum amplitude, which was assumed to be *ω_r_* = 47.91 rad/s. This value is approximately 8% lower than the natural frequency *ω_o_*. This indicates that certain nonlinearities occur at large vibrations. This suggests a weak softening phenomenon. Second, the vibration characteristics of both beams should be nearly identical. However, slight differences visible in Figure 7a and Figure 8a may be attributed to structural differences between the MFC-P1 and MFC-P2 patches, as well as minor variations in the amount of adhesive applied during patch bonding. These factors likely caused the observed small discrepancies in resonance behavior. Furthermore, the load resistance does not significantly affect the beam’s mechanical response; the vibration amplitude curves nearly overlap. This suggests that the electrical load resistance has a negligible influence on the mechanical subsystem dynamics [14]. Lastly, the voltage *U_R_* across the resistor depends on the load resistance. For the same beam vibration amplitude, higher load resistances produce larger voltage outputs.

**Figure 9:**
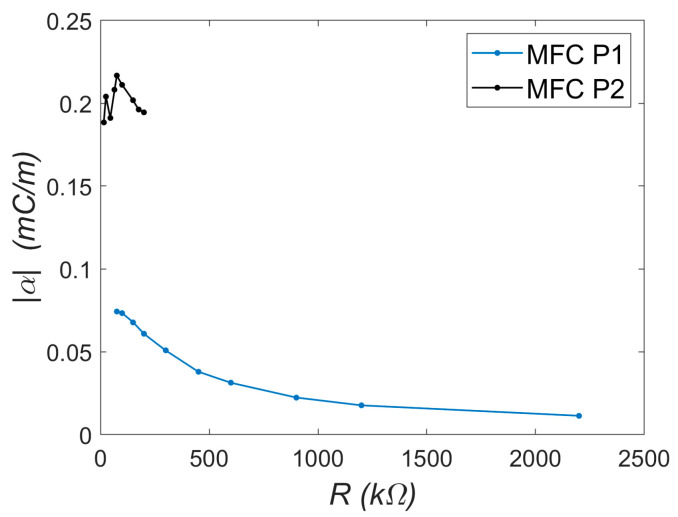
Experimental dependence of the coupling coefficient *α* on load resistance *R*.

Next, the electromechanical coupling coefficient α was determined from Equation (9). Calculations were performed using maximum amplitude values at ω = ω_r_ = 47.91 rad/s. The necessary relative vibration amplitudes *ẅ_o_* were obtained from Equation (12). The resulting acceleration amplitudes were: 143.88 ms^−2^ (corresponding to displacement amplitude *w_o_* = 0.0627 m) for MFC-P1 and 127.58 ms^−2^ (displacement amplitude 0.0556 m) for MFC-P2. These displacement amplitudes correspond to approximately 20% of the beam length: 0.0627/0.316 = 0.198 for P1 and 0.0556/0.316 = 0.176 for P2. Such values indicate that large-amplitude beam vibrations were analyzed in this case. The capacitance *C* of the piezoelectric elements, needed for calculations, was measured using the AX-18B Axiomet multimeter as 7.14 nF for MFC-P1 and 185.64 nF for MFC-P2. Using Equation (9), the dependence *α* = *f*(*R*) was derived and is presented in Figure 9.

The trends observed in Figure 9 are noteworthy. For the P2 element, the coefficient α remains approximately constant at around 2 × 10^−4^ C/m. This trend was determined over a narrow resistance range due to the voltage approaching its operational limit (approximately −60 V during the periodic signal). In contrast, the trend for the P1 element differs: the maximum α value occurs at low load resistances (about 0.75 × 10^−4^ C/m at 75 kΩ) and decreases with increasing resistance (to approximately 0.114 × 10^−4^ C/m at 2200 kΩ). This suggests that while the P2 element is potentially more effective, its usable range is limited. In the present study, (Figure 8b), the P2 system operates near a critical case with maximum energy recovery (periodic voltage amplitude close to −60 V). For the P1 system (Figure 7b), similar voltage levels correspond to about 10% of its lower voltage limit (−500 V). Consequently, the system with MFC-P1 can recover energy from vibrations approximately ten times larger than the system with MFC-P2. Generally, if the electromechanical model is accurate, the coefficient α should be constant. The observed nonlinear behavior may result from large beam deformations, necessitating nonlinear beam theory and higher-order deformation terms [15]. Although vibration amplitudes of both beams are similar (Figure 7a and Figure 8a), the trends *α* = *f*(*R*) differ substantially. If Equation (5) is transformed into the following form:

(13) $$RC\dot{q}+q=\alpha w.$$

In this equation, it can be seen that its right-hand side is approximately the same for the case with element P1 or P2, even when expressed in the form *α*(*w*)*w*. This suggests the presence of nonlinearities in the electrical domain, likely associated with capacitance *C*. Generally, the capacitance can be calculated from the relationship [16]:

(14) $$C=\frac{\varepsilon_{d}A}{h},$$

where

- *ε_d_*—dielectric constants;
- *A*—electrode surface area;
- *h*—distance between electrodes.

Deformation of the MFC element may cause a change in capacitance *C*(*w*), e.g., by affecting the distance *h.* This distance is defined differently for MFC-P1 and MFC-P2: it corresponds to the segment length in P1 and the element thickness in P2. The elongation of the MFC patch directly affects the electrode spacing in the d33 effect (P1), while for the d31 effect (P2), elongation indirectly influences the patch thickness (and thus electrode distance). Another possible source of additional nonlinear capacitance in the system may come from the so-called resistive capacitor [17]. In this approach, the model shown in Figure 1 should be modified by connecting several elastic-sliding elements in parallel with the inductance *L* in the electrical subsystem. The capacitance variation is primarily associated with the electric coordinate *C*(*q*). The effect of the capacitance variation will be analyzed and modeled in future studies.

### 3.3. Summary of Comparative Studies

This subsection provides a concise summary of the results obtained from TEST 1 and TEST 2. The key parameters identified during the experiments are presented in Table 1. Values that are more favorable from a practical (user) perspective are highlighted in bold. The MFC-P2 element shows superior performance in columns 4 and 5, indicating higher electromechanical efficiency. In contrast, the MFC-P1 element outperforms in columns 6 and 7, which refer to its broader range of applicability—specifically, its ability to generate larger structural displacements or to harvest energy from higher-amplitude vibrations. These differences stem from the distinct piezoelectric effects employed in each element type and from structural differences, most notably reflected in the capacitance values (column 3). In conclusion, the selection of a suitable MFC type should be based on the specific requirements of the target application, particularly whether efficiency or operational range is prioritized.

## 4. Study of a Cantilever Bistable Shell with an Embedded MFC Patch

**Figure 10:**
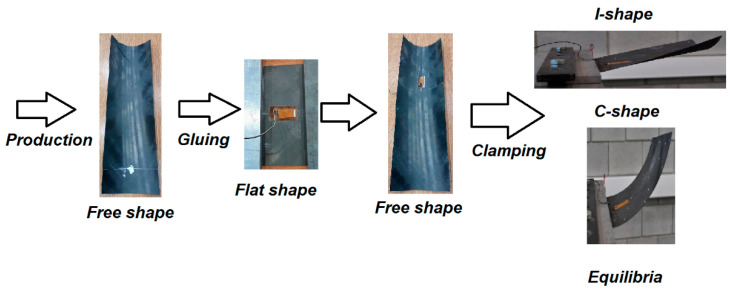
Transformation path of the shell from manufacturing to clamping.

This section presents an investigation of a different structure incorporating an MFC element—a bistable shell. As discussed in the previous chapter, the type of MFC element should be selected according to the characteristics of the host structure. In this case, the shell undergoes large deformations (strains), primarily due to two factors. First, a change in the equilibrium state results in a significant variation in strain—a persistent and substantial strain exists between the two stable configurations. Second, additional strain occurs during dynamic excitation, especially when snap-through behavior is induced. Considering these factors, the P1-type MFC element was deemed more suitable for this application. Additional information on this choice is presented in the free vibration description. Figure 10 shows the sequence of key states of the shell, from manufacturing to final installation.

**Figure 11:**
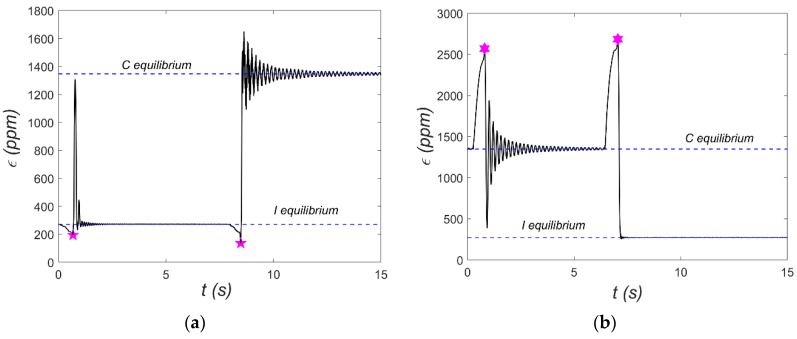
Free vibrations initiated from: (**a**) I-equilibrium; (**b**) C-equilibrium.

Further details regarding the geometry, materials, and fabrication process can be found in [10]. This paper focuses on aspects related to the selection and integration of the MFC patch. In brief, an eight-layer carbon-fiber shell with the stacking sequence [45/-45_2_/45/-45/45_2_/-45] was manufactured using an autoclave process. The shell features variable curvature, radius ranging from 0.07 m to 0.114 m. After trimming to its final dimensions, the shell exhibited a free shape. Next, the MFC patch (model M2814-P1) was bonded to the shell. A small patch size was intentionally selected so as not to suppress the bistability and snap-through effect. Because bonding a patch to a curved surface poses difficulties, the shell was temporarily flattened during the gluing process. After the adhesive cured and the shell was released, it adopted a new free shape. Although the new geometry was only slightly altered compared to the original, residual strains developed in the MFC patch due to bonding in the flattened state. These pre-existing strains were disregarded during testing. A strain gauge was installed on the opposite side of the shell near the center of the MFC patch. It was zeroed (0 ppm) in the shell’s new free state. The shell was then clamped to form a cantilever with an effective length of 0.018 m. Under these boundary conditions, the structure exhibited two stable equilibrium configurations, hereafter referred to as the I-shape and C-shape (Figure 10). Free vibration tests were carried out around each configuration to illustrate the dynamic characteristics of both equilibria (see Figure 11).

The strain gauge mentioned earlier was used to measure the shell’s dynamic response—specifically, the strain *ε* at a single location. Figure 11 includes horizontal dashed lines indicating the strain gauge readings corresponding to the two stable equilibria: for example, *ε* = 1350 ppm in the C-equilibrium state. It is important to refer to the manufacturer’s data [7], which specifies the following:

- Linear–elastic strain limit: 1000 ppm (tensile);
- Maximum operational strain limit: <4500 ppm (tensile).

Achieving snap-through requires much higher deformation—typically at least 2500 ppm—which may lead the MFC patch to exhibit nonlinear behavior.

**Figure 12:**
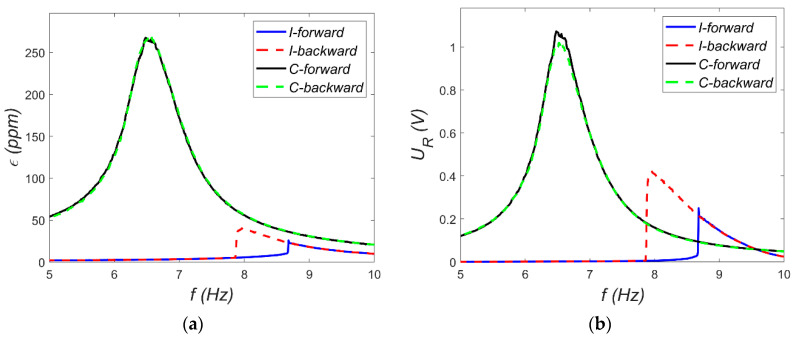
Resonance curves for *a_s_* = 0.2 g: (**a**) strain gauge signal; (**b**) voltage across the harvesting resistor.

**Figure 13:**
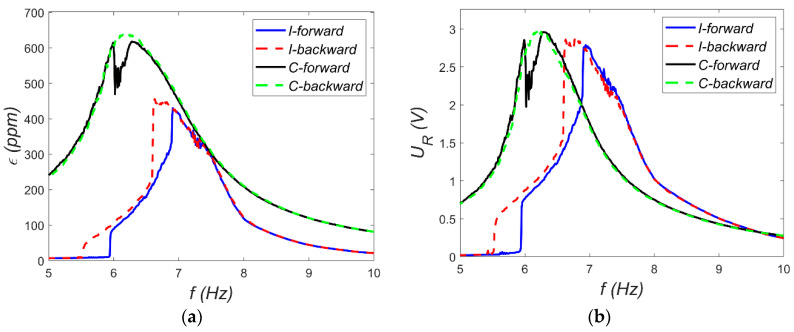
Resonance curves for *a_s_* = 0.8 g: (**a**) strain gauge signal; (**b**) voltage across the harvesting resistor.

During free vibration tests, the shell was deflected from each equilibrium to initiate oscillations. These initial conditions are marked with asterisks in Figure 11. For both equilibrium states, two test cases were presented: one without snap-through and another where snap-through occurred. These tests also reveal the nature of vibrations around each equilibrium. Vibrations around the C-equilibrium are symmetric and resemble the response of a linear oscillator with low viscous damping. It is worth noting that this type of vibration is associated with cyclic extension and contraction of the MFC patch relative to strain in C-equilibrium. In contrast, vibrations around the I-equilibrium are distinctly different, resembling the response of systems with impact dynamics (e.g., a ball bouncing on a surface). Once again, transferring this to the deformation of the MFC patch, it experiences slight elongation and considerably greater compression compared to its I-equilibrium. Overall, the data in Figure 11 show significant strain gauge deformations relative to the desired deformation range of the MFC patch. Another issue is the sign of the strains—for the MFC patch, the strain sign is opposite to that of the strain gauge. As a result, the MFC element is permanently compressed when analyzed relative to its free (unloaded) state. In the structure of P2, patches are additional metal layers between the electrodes and the piezoelectric material, which can cause damage to the structure under large deformation. Therefore, the M2814-P1 element without these additional metal layers was applied. To better investigate the shell’s dynamic characteristics, additional tests were carried out using a Tira 59335 electrodynamic shaker (TEST 2), where the MFC patch served as an energy harvester. In these tests, kinematic excitations with different excitation amplitudes *a_s_* were applied, and frequency *f* sweeps from 5 to 10 Hz were conducted in both forward and backward directions. The harvester load resistance was set to *R* = 400 kΩ. Resonance characteristics for selected excitation levels are shown in Figure 12 and Figure 13. These characteristics were recorded using Scadas Mobile analyzer and Simcenter TestLab v2021 software with a harmonic estimator [18]. The resonance curve for C-mode vibrations displays the expected shape for a nearly linear system. For example, the shift of the resonance point—green series in Figure 12 and Figure 13—is about 0.4 Hz, which is about 6%. This can be considered a weak softening phenomenon. However, at *a_s_* = 0.8 g, a sudden amplitude jump and local distortion of the curve occur near 6 Hz (Figure 13), indicating a snap-through event. This phenomenon corresponds to a transition from C-mode to I-mode vibrations and vice versa. The harmonic estimator struggles to accurately determine amplitude during such nonlinear transitions. The resonance behavior for I-mode vibrations is significantly more complex. At low excitation (*a_s_* = 0.2 g), a pronounced softening effect is observed. Even with small excitations, it is difficult to estimate the value of the natural frequency for this mode. At higher excitation (*a_s_* = 0.8 g), the amplitude jump shifts toward lower frequencies, and a double vertical jump is visible during both forward and backward frequency sweeps—indicative of rich nonlinear dynamics. For such complex dynamics, the shape of the backbone curve is ambiguous.

Applying the electromechanical lumped model from Section 2 to the bistable shell would require a major revision of the mechanical subsystem to account for strong nonlinearities. Capturing the asymmetric bistable behavior requires, at a minimum, a Duffing-Holmes-type model [19]. However, to fully represent the local dynamic variations observed, a higher-order description is needed. As proposed in [20], a fifth-order polynomial may be used to approximate the nonlinear restoring forces more accurately. Due to the complexity of the dynamic behavior, this study focused on estimating the electromechanical coupling coefficient *α* using the relationship defined in Equation (9)—TEST 2, which does not take into account the mechanical subsystem’s properties. The following parameters and assumptions were used:

- Excitation frequencies corresponding to natural frequencies: 6.5 Hz (C-mode) and 9.1 Hz (I-mode);
- Capacitance of the M2814-P1 patch: *C* = 1.176 nF (measured);
- Load resistance: *R* = 400 kΩ;
- Resistor voltage amplitude *U_R_* and strain *ε* were taken from the resonance characteristics.

**Figure 14:**
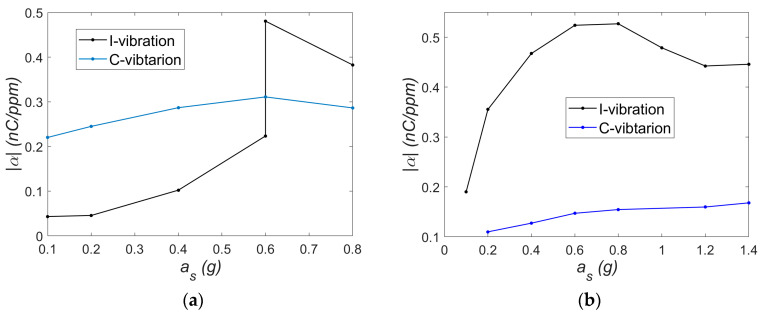
Electromechanical coupling coefficient *α* as a function of excitation amplitude *a_s_* for (**a**) *f* = 5.5 Hz and (**b**) f = 9.1 Hz.

Instead of displacement amplitude *w_o_*, strain *ε* was used in the calculations. The resulting values of *α* as a function of excitation level *a_s_* are shown in Figure 14.

Analyzing the trends *α* = *f*(*a_s_*), it can be observed that near the resonance in the I-mode, the coupling coefficient α is higher for the I-mode vibration (Figure 14b). Beyond the absolute values, a noticeable difference is seen in the shapes of the curves corresponding to I- and C-mode vibrations. For C-mode vibrations, the trend exhibits relatively low variability and could, in a rough approximation, be considered constant. It should be noted that the behavior of the mechanical subsystem in this mode is approximately linear. The nature of vibrations in the range of small and large oscillations may vary slightly, which results in a relatively small change in the variability of the α coefficient. In contrast, such simplification would not be justified for the I-mode, where the coupling coefficient varies more significantly with excitation. However, near the resonance in the C-mode, the *α* coefficient is initially higher for the C-mode vibration (Figure 14a). At an excitation level of *a_s_* = 0.6 g, two stable solutions coexist. A transition (jump) to a higher amplitude response in the I-mode alters the relationship between the *α* values for the I- and C-modes. The trends obtained at a constant load resistance suggest that the coupling coefficient *α* is affected by nonlinearities. In general, mechanical I-mode vibrations exhibit much stronger nonlinear effects compared to case C-mode vibrations. Therefore, there is a clear relationship between the nonlinearities in the mechanical subsystem and the nonlinear behavior of the electromechanical coupling coefficient. In this system, the MFC patch undergoes significant deformation, which may influence its capacitance and contribute to the nonlinear behavior discussed in the previous section. It should also be noted that in this study, strain measurements were taken at a single point, whereas energy harvesting occurs over the entire active surface of the MFC. If the strain distribution across the MFC area changes significantly with vibration amplitude, local strain gauge readings might not accurately represent the overall deformation, potentially affecting the observed trend shapes in Figure 14. Furthermore, according to some researchers, time and temperature effects alone can influence the *α* coefficient in the d33 mode [21], which should be taken into account in future studies.

## 5. Conclusions

The primary objective of this study was to answer two questions: which MFC element—P1 or P2—is more effective, and which of these elements is more suitable for energy harvesting from the mechanical vibrations of bistable shells. Both questions were addressed to a significant extent. Section 3 presented a detailed comparative analysis of the P1 and P2 elements, and the results were summarized in Section 3.3. The answer to the first question is not straightforward: the P2 element demonstrates higher electromechanical effectiveness (about twice as large), while the P1 element offers a broader operational range (about twice as large during activating displacements and quintuples as large when recovering energy). Therefore, selecting the appropriate MFC type must be based on the specific requirements and characteristics of the target application. This principle was applied to the bistable shell system. After careful analysis, a compact P1-type patch was chosen. The key challenge in this case was the high level of deformation (strain), which approaches the operational limits (2500 ppm > linear limit = 1000 ppm) of the MFC material. Despite prolonged operation under these conditions, the P1 element remained functional and undamaged. The experimental results confirmed that the P1 patch can successfully harvest energy from mechanical vibrations around both stable equilibria of the bistable shell. During the research, it became evident that the proposed electromechanical model has some limitations and thus it will be extended in future work. Both the coupling mechanism and the electrical circuit were assumed to be linear. However, the obtained results indicate that nonlinear terms should be considered. The effect of the MFC patch capacitance requires further investigation—particularly in the case of element P1, where additional nonlinearities may need to be taken into account. It is also observed that the correction of the electromechanical coupling coefficient is needed, especially in situations where the mechanical subsystem exhibits significant nonlinear behavior (strong nonlinearities observed by a larger variation in electromechanical coupling). These improvements are planned for future work.

## Figures and Tables

**Table 1:** Comparison of selected parameter values for MFC-P1 and MFC-P2 elements.

Symbol	Value	Capacitance *C* (nF)	Parameter |*α*_1_| (ms^−2^V^−1^)	Parameter |*α*| (C/m)	Actuator |*w_max_* − *w_min_*| (mm)	Harvester |*U_Rmax_*/*U_downlimit_*| (-)
M8528-P1	*V*_1_	7.14	25.07 × 10^−3^	<0.75, 0.114 > × 10^−4^	**17**	**0.1202**
M8528-P2	*V*_2_	185.64	**50 × 10^−3^**	**≈2 × 10^−4^**	8	0.8464
-	Ratio *V*_1_/*V*_2_	0.0385	0.5014	<0.375, 0.057>	2.125	0.1412

## Data Availability

The original contributions presented in this study are included in the article. Further inquiries can be directed to the corresponding author.
